# Metabolic Rate Regulates L1 Longevity in *C. elegans*

**DOI:** 10.1371/journal.pone.0044720

**Published:** 2012-09-06

**Authors:** Inhwan Lee, Amber Hendrix, Jeongho Kim, Jennifer Yoshimoto, Young-Jai You

**Affiliations:** 1 Departments of Biochemistry and Molecular Biology, Virginia Commonwealth University, Richmond, Virginia, United States of America; 2 Department of Biological Science, Inha University, Incheon, Korea; 3 Department of Internal Medicine, University of Michigan, Ann Arbor, Michigan, United States of America; VIB & Katholieke Universiteit Leuven, Belgium

## Abstract

Animals have to cope with starvation. The molecular mechanisms by which animals survive long-term starvation, however, are not clearly understood. When they hatch without food, *C. elegans* arrests development at the first larval stage (L1) and survives more than two weeks. Here we show that the survival span of arrested L1s, which we call L1 longevity, is a starvation response regulated by metabolic rate during starvation. A high rate of metabolism shortens the L1 survival span, whereas a low rate of metabolism lengthens it. The longer worms are starved, the slower they grow once they are fed, suggesting that L1 arrest has metabolic costs. Furthermore, mutants of genes that regulate metabolism show altered L1 longevity. Among them, we found that AMP-dependent protein kinase (AMPK), as a key energy sensor, regulates L1 longevity by regulating this metabolic arrest. Our results suggest that L1 longevity is determined by metabolic rate and that AMPK as a master regulator of metabolism controls this arrest so that the animals survive long-term starvation.

## Introduction

In nature, animals often face long-term starvation without knowing when the next meal will be. How they survive long-term starvation, how long they can survive or how long-term starvation affects the animal after it recovers from starvation, however, are not clearly understood. When hatched in the absence of food, the first stage larvae (L1s) of *C. elegans* survive starvation for approximately two weeks. This arrest, called L1 diapause, is distinct from another form of developmental arrest called dauer diapause that occurs at a stage equivalent to the third-stage larva (L3). Whereas the molecular mechanisms of dauer diapause have been intensively studied, those of L1 diapause have not. It has been shown, however, that L1 arrest requires reduced insulin signaling [1], [2], suggesting metabolic rate might be a crucial factor to survive starvation during L1 arrest.

AMPK is activated by a high ratio of AMP to ATP. Once activated, AMPK inhibits anabolic pathways and activates catabolic pathways to maintain a proper level of energy for the cell to survive a low energy state. Many key catabolic pathways such as fatty acid oxidation are activated by AMPK to provide energy. In contrast, many key regulators of anabolic pathways such as mTOR are inhibited by AMPK to reduce energy consumption [3]. In addition, AMPK initiates autophagy, a self-eating process to produce energy, by directly phosphorylating ULK-1, a homolog of atg1 in yeast that initiates autophagy [4]. All these studies show that AMPK functions as a central energy sensor to couple energy status to metabolism in the cell. However, the molecular mechanisms by which AMPK plays roles during prolonged energy deprivation such as long-term starvation at the whole organism level are less understood.

In this study, we show that survival span of arrested L1s, which we call L1 longevity, depends on metabolic rate during starvation; if we increase metabolic rate either by using genetic mutants or by increasing temperature, L1 longevity is reduced. If we decrease metabolic rate, the longevity is enhanced. In addition, after long term starvation, survived animals show post-starvation phenotypes such as organ damage or slow growth, suggesting that L1 arrest has metabolic costs. We also show that AMPK is one of the key regulators of the metabolic arrest in L1 longevity; in the absence of AMPK, worms cannot survive long-term starvation, probably because they fail to maintain proper metabolic rate or energy level during starvation.

## Results

### L1 Survival Span is Regulated by Metabolic Rates

**Figure 1:**
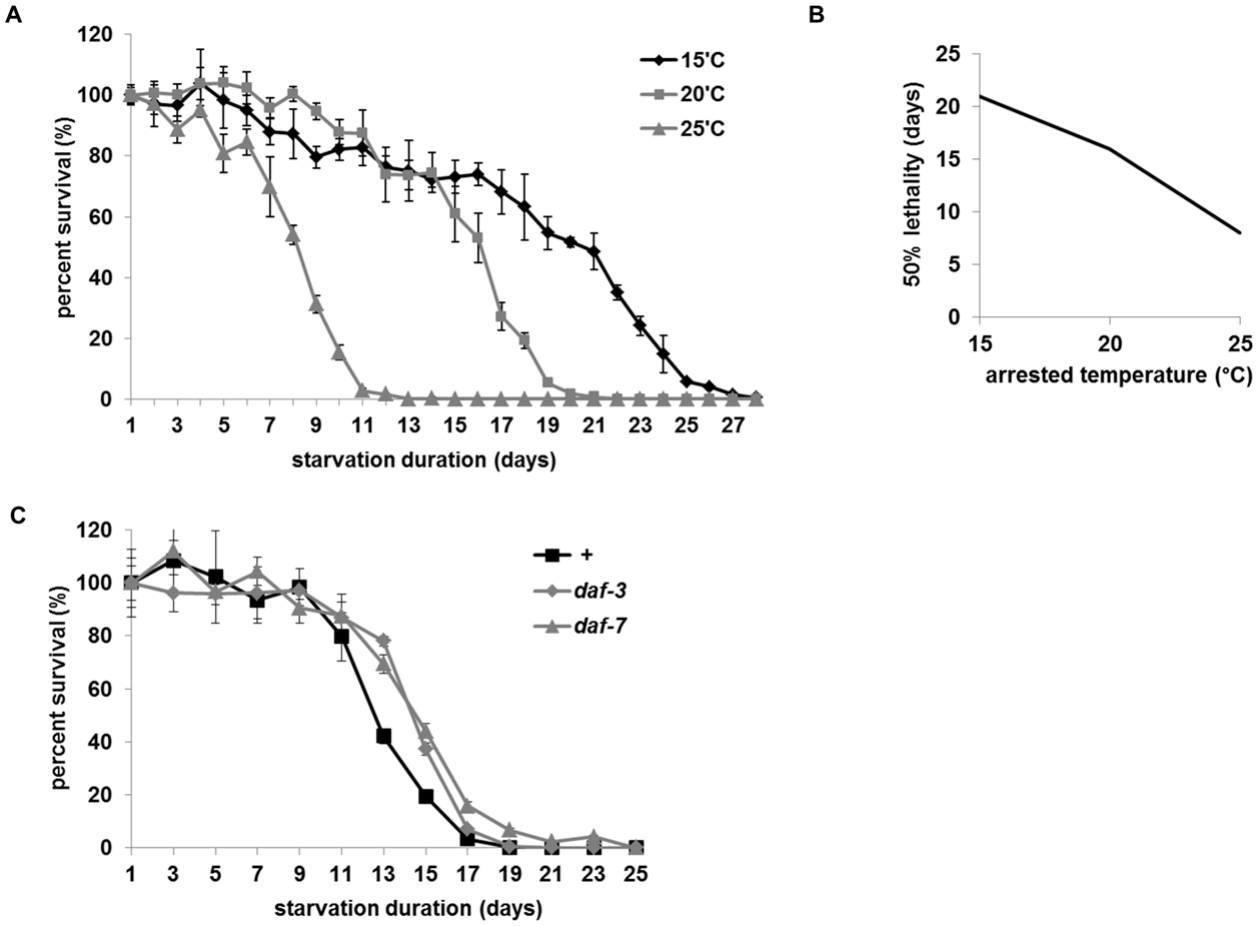
L1 arrest is a starvation response depending on metabolic rates. A L1s arrested at low temperature live longer than those arrested at high temperature. Eggs were collected and hatched without food and L1s were maintained at the indicated temperatures [26]. For all L1 longevity assays, each sample contained approximately 10 worms/µl and each plate was plated with approximately 150–200 worms. Every experiment was repeated at least three times with triplicates (see Materials and Methods). B LT 50 (50% lethality) for worms that arrested at 15°C, 20°C and 25°C. C Life spans of arrested L1s of *daf-7* and *daf-3* mutants tested at 25°C. D Rates of oxygen consumption in wild type, *daf-2* and *daf-16* mutants (**p<0.01, by two way ANOVA). E Rates of oxygen consumption in wild type and *daf-18* mutants (*p<0.05, by two way ANOVA).

Metabolic rate depends on temperature. To test if survival span of arrested L1 is regulated by metabolic rate, we measured the survival span of L1s arrested at three different temperatures: 15°C, 20°C and 25°C. L1s arrested at 15°C have the longest survival span with LT_50_ (50% of lethality) of 21 days, while the L1s arrested at 25°C have the shortest survival span of 8 days. At 20°C LT_50_ = 16 days, showing a clear inverse correlation between survival span of arrested L1s and the temperature at which they were arrested (Figure 1A and 1B). It has been shown that L1 arrest is regulated by the insulin signaling pathway, which alters metabolism and thus regulates adult life span [1], [2], [5]. As previously reported, reduced function mutants of the insulin receptor gene (*daf-2*) show longer survival span of L1s than wild type (Figure S1A, B, C), whereas mutants of PTEN (*daf-18*) and FOXO (*daf-16*), which cannot antagonize insulin signaling and thus could have increased metabolic rates during starvation, show a shorter L1 survival span than wild type (Figure S2).

To validate the difference in metabolic rates in the mutants, we measured the oxygen consumption rates in adults (see Methods). *daf-2* mutants show a reduced oxygen consumption rate whereas *daf-16* mutants show an increased oxygen consumption rate compared to wild type, supporting the inference that the mutations in insulin-FOXO pathways alter metabolic rates (Figure 1D). Many *daf-18* adults are sick (approximately 30%, data not shown). To avoid the variation caused by sick worms, we measured the oxygen consumption rate of *daf-18* when they were L4s and compared it with that of wild type L4s. As shown in Figure 1E, *daf-18* has an increased oxygen consumption rate, also supporting our hypothesis.

Insulin signaling also regulates dauer diapause, another worm developmental arrest. To examine if L1 survival span depends on the dauer formation pathway, we tested two mutants of the TGF β pathway: *daf-7*, which encodes the ligand, and *daf-3*, which encodes a smad transcription factor involved in TGF β signaling. Like *daf-2* mutants, *daf-7* mutants have a higher tendency to become dauers. And like *daf-16* mutants, *daf-3* mutants are defective in dauer formation [6]. When we tested them at 25°C, a non-permissive temperature for *daf-7* mutants, *daf-7* mutants showed a slight decrease in L1 survival span, whereas *daf-3* mutants showed a slight increase in L1 survival span (Figure 1C). This result suggests that the TGF β pathway may play roles in L1 survival span but it functions differently in L1 survival than it does in dauer formation; unlike *daf-16* mutants, the *daf-3* mutant is long-lived, and *daf-7* is not as long lived as *daf-2*. In addition, the difference in the L1 survival spans among wild type, *daf-7* and *daf-3* is very small. Moreover, when we tested *daf-7* mutants in other temperatures (20°C and 22.5°C) there was no significant difference between wild type and *daf-7* mutants in L1 survival span (Figure S3). Therefore our finding suggests that L1 survival span is affected primarily by metabolic rate but not by dauer arrest cues such as absence of TGF β signaling. To distinguish the survival span of arrested L1s from adult life span, we call it L1 longevity.

### Long-term L1 Starvation Damages Gonad and Delays Development during Recovery

**Figure 2:**
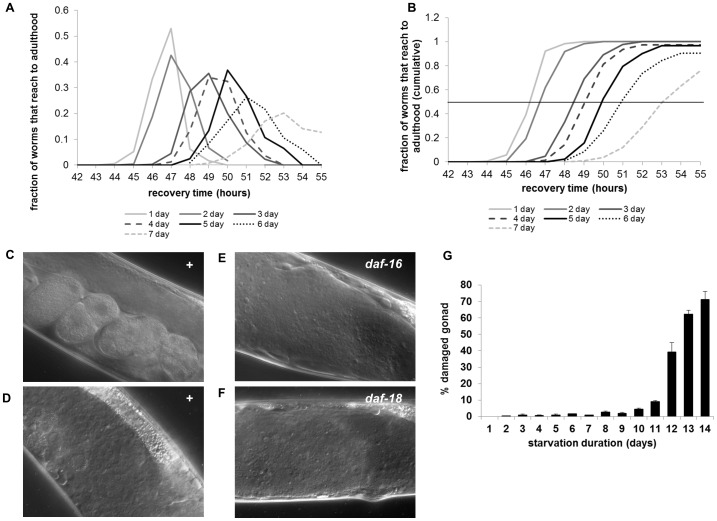
Long-term L1 starvation causes tissue damage and delay in development during recovery. A Fraction of worms that reach adulthood after indicated days of L1 starvation (see Materials and Methods). Approximately 100 worms were used per experiment and each experiment was done with triplicates. B Cumulative fraction of worms that reach adulthood after indicated days of L1 starvation. Line drawn to show the time when 50% worms reach adulthood after indicated hours of refeeding. C DIC (Differential Interference Contrast) image of wild type gonad: the worm was starved for 3 days as an L1 and recovered to grow to an adult. For all experiments to observe gonad defect, approximately 100 worms were used per experiment and each experiment was done with triplicates. D DIC image of wild type gonad: the worm was starved for 9 days as an L1 at 20°C and recovered to grow to an adult at 20°C. No eggs are visible. E–F DIC images of gonads of *daf-16* mutants (E) and *daf-18* mutants (F): worms were starved for 3 days at 20°C as L1s and recovered to grow to adults at 20°C. No eggs are visible. G Percent destroyed gonad increases as L1 starvation continues.

Our results suggest that L1 longevity is regulated by metabolic rate during starvation. To see what starvation does to L1s so as to understand how worms survive it, we examined the recovery of L1s that have been starved for various periods, after giving them food. The longer they starve, the slower the worms develop. When we measured the time point where 50% of worms reach young adults, we found that development was delayed about 1–2 hour(s) for each day of starvation (Figure 2A & 2B) (see Materials and Methods). The appearance of the first young adult is also delayed (Figure 2A). These post-starvation effects on recovery suggest that an extra day of starvation costs developmental delay because the longer starved worms have fewer resources left to maximize their developmental rate during recovery after starvation. After 7 days of starvation, a fraction of worms died or developed too slowly (Figure 2B), suggesting that from this point starvation damaged worms irreversibly, so that some worms could not fully recover. The organ that gets damaged most apparently is the gonad (Figure 2C for 3 day starved and 2D for 9 day starved). The damaged gonad predicted reduced brood size; when we picked L4 worms before they developed the gonad defect then compared the numbers of progeny between worms whose gonads were damaged and worms whose gonads were not, we found worms with damaged gonads had reduced brood size or became sterile (Table S1). The fraction of worms that show the damaged gonad in adults increases as starvation as L1s continues, suggesting that L1 starvation induces the post-starvation phenotype (Figure 2G). When we examined mutants whose metabolism is misregulated during L1 starvation and which have short L1 lifespan, both *daf-16* and *daf-18* showed the same damaged gonad phenotype from as early as 3 days of starvation (Figure 2E and 2F). This suggests that a damaged gonad as post-starvation phenotype can result from misregulation of metabolism during L1 starvation.

### AMPK is Necessary for L1 Longevity

**Figure 3:**
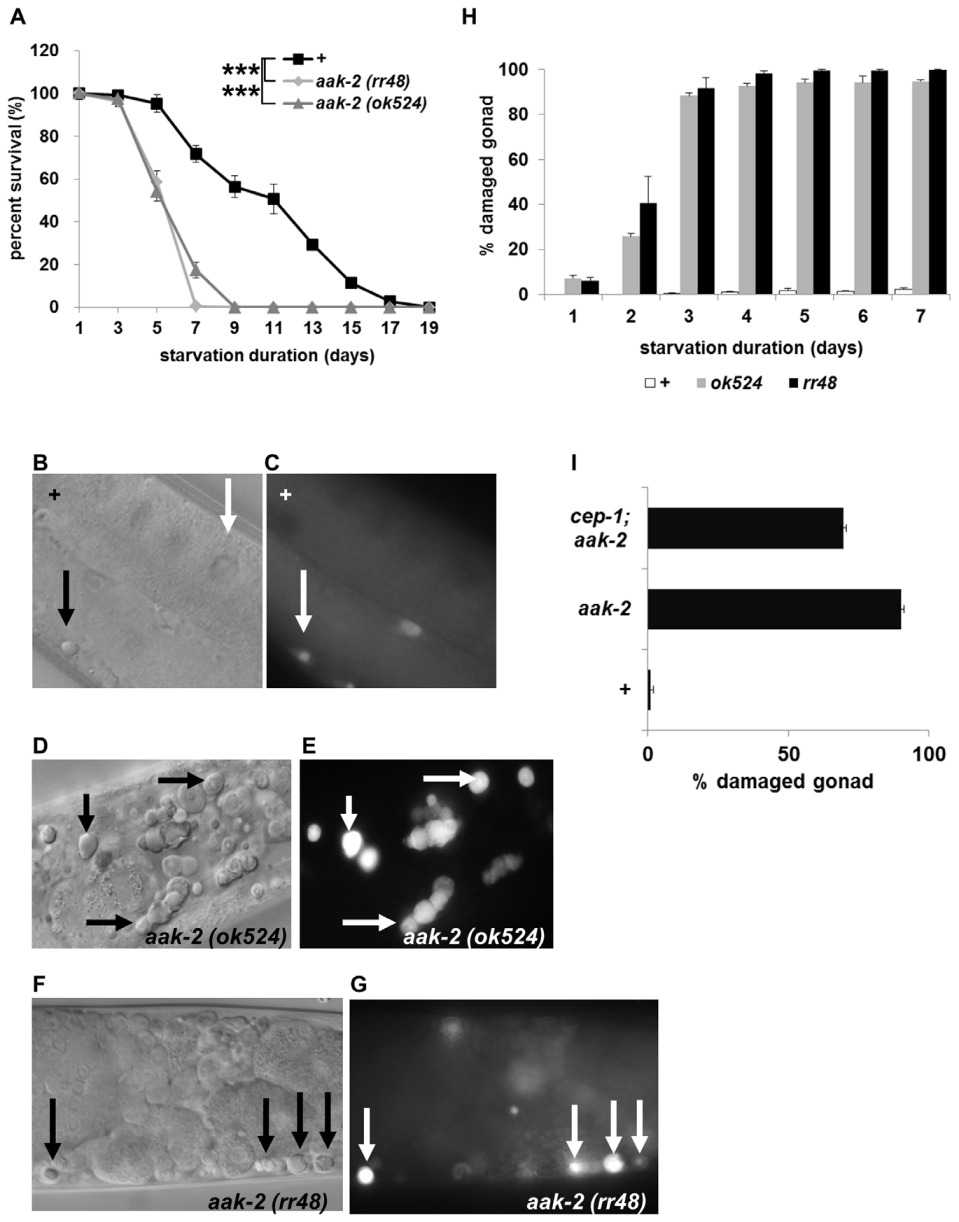
AMPK is necessary for L1 longevity. A Percent survival of wild type (▪) and two independent *aak-2* mutants (*ok524* (gray ▴) and *rr48* (gray ♦)) after L1 starvation at 22.5°C for the indicated number of days. B DIC (Differential Interference Contrast) image of wild type gonad. The black arrow indicates the button like structure of the nucleus of a dying cell. The white arrow indicates an oocyte. C Fluorescent image of the same worm stained with acridine orange to detect cell death. The white arrow indicates the dying cell. D&F DIC images of AMPK mutant gonads. The black arrows indicate dying cells. Gonads are destroyed and no oocytes are visible. E&G Fluorescent images of the same worms stained with acridine orange reveal more dying cells (the white arrows) in the gonads of AMPK mutants. H Percent destroyed gonads in two independent *aak-2* mutants. I After 3 days of L1 starvation at 22.5°C and recovery, *cep-1* mutation partially rescued *aak-2* gonad phenotype.

Because metabolic rate regulates L1 longevity and because AMPK regulates metabolism in a low energy state such as starvation, we focused on L1 longevity of *aak-2* mutants. *aak-1* and *aak-2* are two *C. elegans* genes encoding AMPK α subunits. RNA interference of *aak-1* does not cause an obvious abnormality [7]. In contrast, *aak-2* mutants are short-lived as adults, sensitive to mitochondrial poisoning and defective in arresting germ cell division both in dauers and in L1 diapause, suggesting that *aak-2* is more important than *aak-1* [1], [7], [8]. As previously reported [1], *aak-2* mutants die earlier than wild type during L1 starvation; after 7 days of starvation 90% of them died, whereas only 20% of wild type did (Figure 3A). In addition, when the animals recover from starvation, their gonads are damaged after 3 days of L1 starvation (Figure 3B, 3D and 3F). The gonad damage is most prominent as they become 2 day old adults and is often accompanied by programmed cell death (Figure 3C, 3E and 3G). The fraction of worms whose gonads were damaged increases as starvation continues; after 7 days of starvation over 95% of live worms show damaged gonads and become sterile (Figure 3H). When we counted the number of gonad precursor cells, we found that *aak-2* mutants have more cells than wild type (Figure S4A), confirming that the mutants fail to arrest the cell cycle during starvation and that that might be the reason the mutants have damaged gonads once they become adults. We counted the cells in the gonad after 5 days of L1 starvation following 12 hours of refeeding in order to ensure the identity of the cells we count. To exclude the possibility that the increased cell numbers in gonads is due to faster development in *aak-2* mutants than wild type during 12 hours of refeeding, we measured the growth rate and the gonad development of *aak-2* mutants. Both growth rate and gonad development were delayed in *aak-2* mutants, suggesting that increased cell numbers in *aak-2* mutants are not because of fast development but because of failure of cell cycle arrest during L1 starvation (Figure S4B–D, Table S2).

Because we observed apoptosis in the gonad and because two apoptosis pathways, caspase-dependent and p53 dependent, have been reported in *C. elegans* germ cells, we tested if the cell death in the gonads of *aak-2* mutants are dependent on them. A mutation in *ced-3*, which encodes a *C. elegans* homolog of ICE caspase, does not rescue either *aak-2* cell death in gonads or damaged gonad (data not shown), but a mutation in *cep-1,* which encodes p53, rescues partially (Figure 3I). This shows that cell death in the gonad induced by long-term starvation is partially dependent on p53.

### Down Regulation of Metabolism or Addition of Glucose Enhances *aak-2* L1 Longevity

**Figure 4:**
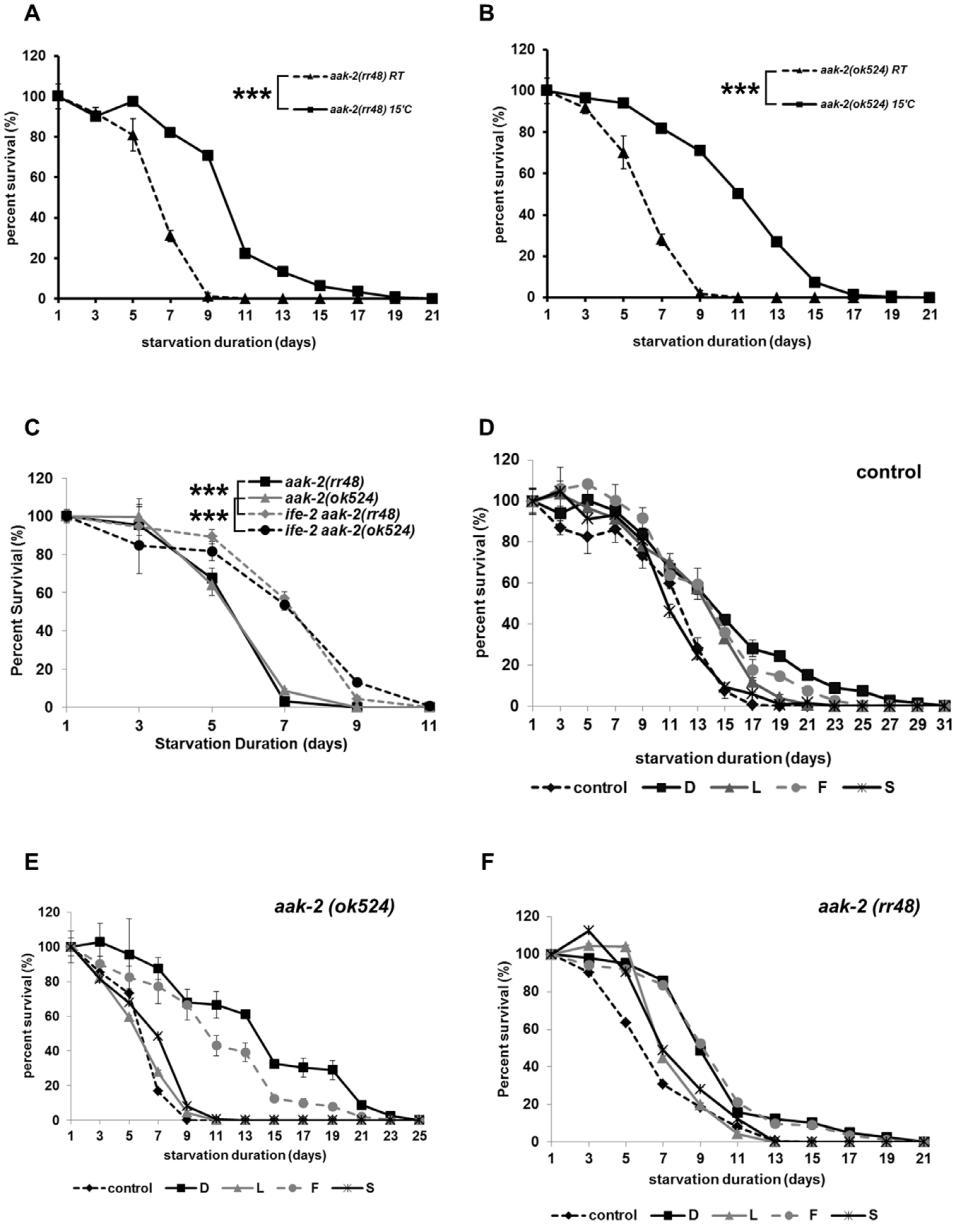
Down regulation of metabolism or addition of glucose enhances *aak-2* L1 longevity. A–B Starvation at 15°C enhances *aak-2* L1 longevity in *aak-2* mutants (A: *rr48,* B: *ok524*), ***p<0.001. C Mutation in *ife-2* enhances *aak-2* L1 longevity, ***p<0.001. D Glucose and fructose (100 mM) slightly enhanced L1 longevity of wild type but sorbitol and L-glucose didn’t. *p<0.001* between control and the wild type treated with glucose or fructose. D: D-glucose, L: L-glucose, F: fructose, S: sorbitol. E–F Glucose and fructose enhanced *aak-2* L1 longevity but sorbitol and L-glucose didn’t. *p<0.001* between control and the *aak-2* mutants treated with glucose or fructose. For each experiment, approximately 100–200 L1s were used. D: D-glucose, L: L-glucose, F: fructose, S: sorbitol.

To survive starvation, animals turn off anabolic pathways to reduce their energy use and turn on catabolic pathways to replenish the energy. If *aak-2* L1s die early because the mutants fail to turn off their anabolic pathways so that they consume stored energy too fast during starvation with higher metabolic rate than wild type, we could enhance *aak-2* L1 longevity by reducing its metabolic rate. We tested this idea in two ways: (1) we arrested *aak-2* mutants at a low temperature (15°C). When we arrested *aak-2* mutants at 15°C, they survived longer (Figure 4A and 4B). (2) We reduced protein synthesis rate by inhibiting the mTOR pathway. Because AMPK directly phosphorylates TORC to inhibit protein synthesis when energy is low, in the absence of AAK-2, *aak-2* mutants might fail to inhibit the mTOR pathway and therefore deplete energy rapidly and die early. Because mutants of *C. elegans* TOR (*let-363*) are larva lethal, we tested an *ife-2* mutant instead. *ife-2* encodes a translation initiation factor 4E and functions downstream of mTOR [9]. *ife-2* mutants have increased adult life span [9] as well as L1 longevity (Figure S5) compared to wild type. Introducing an *ife-2* mutation into *aak-2* mutants enhances *aak-2* L1 longevity (Figure 4C). Therefore both means of lowering metabolic rate improved the short L1 longevity of *aak-2* mutants, suggesting that *aak-2* is necessary for normal L1 longevity and works by reducing energy consumption during starvation.

To further test if lack of energy causes short L1 longevity in *aak-2* mutants, we added glucose (100 mM), which can be easily converted to energy during L1 starvation. Glucose increases the L1 longevity of wild type by only 2 days (Figure 4D) suggesting that the beneficial effect of glucose is occluded by the already maximum longevity of wild type. However glucose restores normal L1 longevity to one of two *aak-2* mutants and partially restores it in another, strongly suggesting that the short L1 longevity of *aak-2* is because of rapid depletion of energy during starvation (Figure 4E and 4F). L1 longevity of *aak-2* was also enhanced by the addition of fructose as an alternate energy source (Figure 4E and 4F). That two different types of sugars rescued *aak-2* mutants suggests it is the energy from sugars but not a specific type of sugar that rescued *aak-2* mutants. To exclude the possibility that glucose acts as a sensory cue rather than an energy source, we treated worms with L-glucose, which has little energy value but is indistinguishable in taste sensation [10]. It doesn’t rescue the short L1 longevity of *aak-2*, confirming that glucose rescues short L1 longevity of *aak-2* by providing energy (Figure 4E and 4F).

### L1 Starvation Activates AAK-2 in PAR-4, LKB Dependent Manner

**Figure 5:**
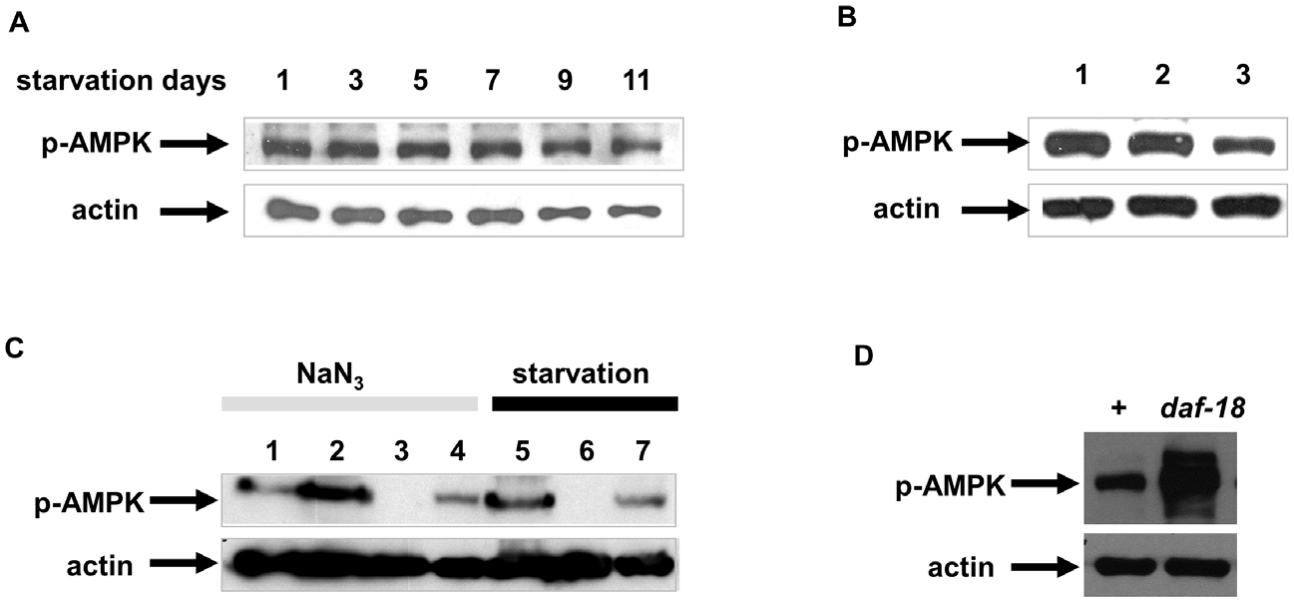
L1 starvation activates AAK-2 partially depending on PAR-4, *C. elegans* LKB1. A. L1 starvation activates AMPK. L1s were starved for indicated days and collected to be prepared for Western blot assay (see Materials and Methods). B. Refeeding for one hour after 3 days of starvation reduced activation of AAK-2. Lane 1∶1 day starved L1s, Lane 2∶3 day starved L1s, Lane 3: L1s were starved for 3 days and refed for 1 hour. C. AAK-2 is phosphorylated in response to starvation and mitochondrial poisoning with NaN_3_ in a partially PAR-4 dependent manner. Lane 1: wild type adults placed on unseeded NGM plates for 6 hours (control), Lane 2: wild type adults treated with 1 mM NaN_3_ for 6 hours on unseeded NGM plates, Lane 3: *aak-2* mutants (*ok524*) treated with 1 mM NaN_3_ for 6 hours, Lane 4: *par-4* mutants treated with 1 mM NaN_3_ for 6 hours, Lane 5: wild type adults starved for 24 hours in M9, Lane 6: *aak-2* mutants (*ok524*) starved for 24 hours, Lane 7: *par-4* mutants starved for 24 hours. D. Starvation-induced AAK-2 phosphorylation is *daf-18* independent. Both wild type and *daf-18* mutants were starved for 2 days to measure phosphorylation of AAK-2. Mutation in *daf-18* doesn’t decrease but instead increases AAK-2 phosphorylation by starvation, suggesting AAK-2 phosphorylation does not require DAF-18 activity.

To test if AAK-2 can be activated during L1 starvation, we measured the activation by detecting its phosphorylation at the threonine residue 172, using a phospho-specific antibody for AMPK (see Materials and Methods). AAK-2 is activated throughout starvation from day 1 (Figure 5A). To collect enough L1s for the western blot assay, we harvest eggs and synchronize them at L1 by starving them overnight [11]. Because this method includes overnight starvation, we could not test the phosphorylation level of AMPK in well-fed L1s. Instead, after 3 days of L1 starvation, worms were refed for one hour. The activation of AAK-2 was reduced significantly by refeeding, suggesting that starvation induced AAK-2 activation (Figure 5B). Based on the finding that 1 mM NaN_3_ significantly decreases viability of *aak-2* mutants [7], we treated adult worms with NaN_3_ (1 mM) for 6 hours and were able to observe AAK-2 activation (Figure 5C, lane 2). When synchronized young adult worms were starved for 24 hours, AAK-2 was also activated (Figure 5C, lane 5). The activation of AAK-2 either by mitochondria poisoning or by starvation was abolished in *aak-2* mutants, confirming that the band was AAK-2 (Figure 5C, lane 3, 6 respectively). We could not detect a band corresponding to AAK-1, probably because the peptide sequence in the region around Thr 172 is not conserved between AAK-1 and AAK-2 (data not shown).

To be fully activated, Thr172 on AMPK has to be phosphorylated by its upstream kinases [12]. Thus we next asked if the AAK-2 activation is dependent on its known upstream kinase LKB1 [13], PAR-4. PAR-4 is essential for embryogenesis in *C. elegans* [14]. We used a temperature sensitive allele (*it47*) and grew the worms at 23°C (a non-permissive temperature) from L1 [15]. None of the L1 progeny produced viable eggs when they grew to adults, confirming that there was no functional PAR-4 left (data not shown).

**Figure 6:**
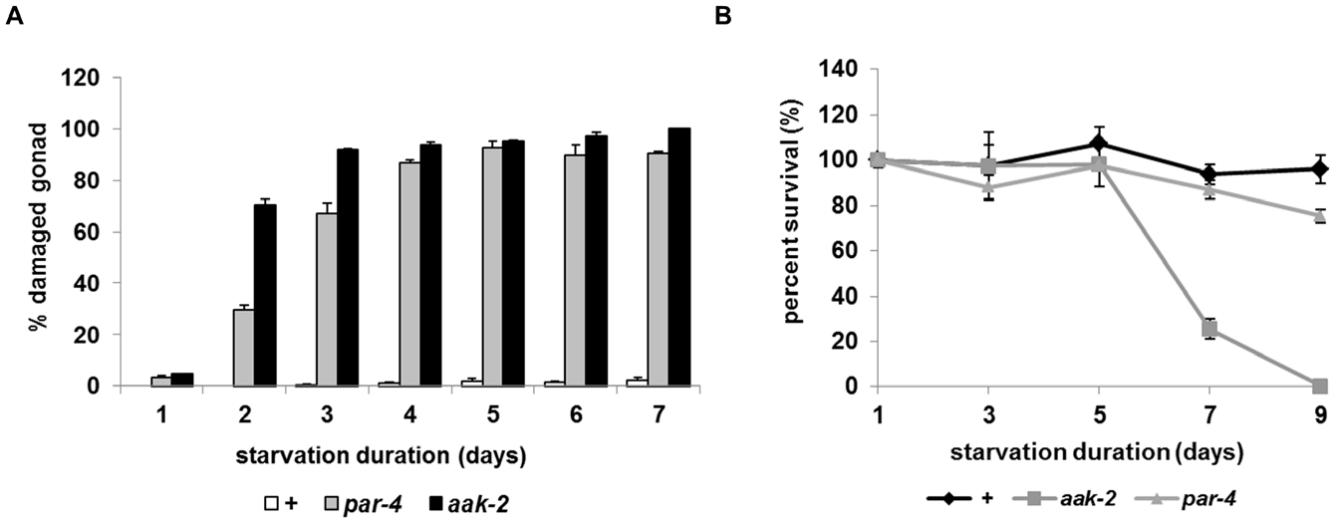
*par-4* partially phenocopies starvation-induced phenotype of *aak-2*. A. Percent damaged gonad of wild type and *par-4* mutants after L1 starvation for the indicated days. B. Percent survival of wild type (♦), *aak-2* (gray ▪) and *par-4* (gray▴) mutants after L1 starvation for the indicated days.

When we tested if starvation or mitochondrial poisoning can cause AAK-2 phosphorylation in *par-4* mutants, we found that for both stresses phosphorylation was reduced in *par-4* mutants (Figure 6C, lane 4 for mitochondrial poisoning and lane 7 for starvation). However, some AAK-2 phosphorylation still remains, suggesting that AAK-2 activation by starvation or by mitochondrial poisoning is only partially dependent on PAR-4.

Because L1 longevity of *daf-18* mutants is also short, we examined if PTEN genetically interacts with AMPK. Because both mutants have short L1 longevity, we tested if the absence of PTEN activity in *daf-18* mutants abolishes AMPK phosphorylation and therefore kills *daf-18* worms in L1 starvation. When we measured AMPK phosphorylation in PTEN mutants, however, AAK-2 phosphorylation by starvation is intact and even increased in *daf-18* mutants (Figure 5D). This over-activation of AMPK can suggest that in PTEN mutants, insulin signaling cannot be reduced during starvation and ATP is depleted faster than in wild type, which in turn activates AMPK more. Supporting this notion, introducing a *daf-2* mutation partially rescues *aak-2* mutants’ short L1 longevity (Figure S6). Slowing down insulin pathway signaling partially rescues *aak-2* mutants probably by reducing the basal metabolic rate of *aak-2* mutants.

### Mutation of *par-4*, which Encodes LKB1, Partially Phenocopies the Starvation-induced Phenotype of *aak-2*

Next we examined if starvation also induces the gonad destruction phenotype in *par-4* mutants. The fraction of worms showing destroyed gonads increases as starvation continues (Figure 6A). Unlike the gonad destruction and sterility phenotypes, viability of *par-4* mutants does not decrease as rapidly with starvation as it does in *aak-2* mutants, suggesting that the short L1 longevity of *aak-2* might not be solely dependent on *par-4* (Figure 6B). This partial resemblance (but not perfect phenocopy) in phenotypes between *aak-2* and *par-4* has been also reported in germ cell arrest in dauers; *par-4* mutants mimic the *aak-2* mutant phenotype only partially [8]. Our data that starvation causes AAK-2 phosphorylation in the absence of PAR-4 and that the starvation-induced phenotypes of *aak-2* mutants and *par-4* mutants partially resemble each other suggest that other upstream kinase(s) may function in the *aak-2*-dependent survival long-term starvation pathway.

## Discussion

Animals respond to starvation in a complicated but systematic way [16]. Hatched without food, *C. elegans* L1s survive for approximately two weeks. Recent studies show that L1 arrest is regulated by the insulin pathway and that it is an active process of responding to stress during which worms are prepared for growth once food becomes available [1], [17]. In addition, L1 survival span can be extended without promoting further development into L2 by adding chemicals such as ethanol that can provide a carbon and energy source [17], [18]. These observations strongly suggest that the arrested L1 stage could be another stress-resistant developmental stage, which is mainly influenced and regulated by signals and available sources of nutrients. Moreover Zhang et al showed that during L1 arrest, the expression of genes in the insulin pathway is repressed by *mir-71*, a micro RNA, showing that active and specific molecular mechanisms are necessary for worms to be arrested as L1 [19]. In this study we showed that the L1 survival span is also regulated by metabolic rate. Low metabolic rate allows L1s to survive longer than worms with high metabolic rate; L1s arrested at lower temperature survive longer than those arrested at higher temperature. Also, down-regulating the insulin pathway, which reduces metabolic rate, allows worms to survive L1 starvation longer. In addition, L1 starvation damages worms. Long-term starvation not only reduces survival, but also delays the recovery of surviving worms. As for the molecular mechanisms, we showed that AMPK functions as a master energy sensor to regulate a starvation response at the whole organism level in *C. elegans*.

AMPK plays central roles in development, cell cycle arrest, and energy homeostasis in mammals [20]. These functions are conserved for worms to live a normal life span, to resist stress, to maintain germ cell integrity and to arrest the cell cycle under energy-deprived conditions [1], [7], [8], [21]. In addition, we showed that lack of functional AAK-2 during starvation causes sterility when the worms recover on food and become adults. We found that, as previously reported during L1 arrest, in *aak-2* mutants certain cells including germ line precursor cells fail to arrest the cell cycle and instead keep dividing [1]. We speculate that this failure to arrest the cell cycle in *aak-2* mutants during starvation eventually contributes to gonad damage accompanied by cell death in a p53 dependent manner. It is an intriguing question, however, how the failure of starvation-induced cell cycle arrest in *aak-2* mutants causes damage to the gonad at a later stage of development.

AMPK requires phosphorylation for its full activation. Among three known upstream kinases we found only mutants of *par-4*, a homolog of LKB [14], partially mimic the sterility phenotype of *aak-2* mutants. However, since *par-4* mutants didn’t completely abolish AAK-2 phosphorylation during starvation or mitochondrial poisoning, more studies are needed to find upstream kinases other than PAR-4 that activate AMPK during starvation.

In conclusion, our study shows that metabolic rate during L1 starvation is a crucial factor in determining how long the animal can survive starvation. In addition, our study also demonstrates that there are post-starvation effects such as organ damage or slow growth. By providing evidence that L1 longevity is regulated by metabolic rate, our study will help to address other interesting questions such as what the genetic components of L1 longevity regulated by metabolic rate are. Also our study shows that L1 arrest probably uses molecular mechanisms that are not exactly the same as those of dauer arrest. Finally, we suggest an essential role of AMPK in whole organism starvation survival. The lethality and sterility phenotypes of AMPK mutants can be used to find molecules that interact with the AMPK signaling pathway.

## Materials and Methods

### General Methods for Culturing Worms and Strains

Worms were cultured and handled as described previously [22] with the following modifications: worms were routinely grown on NGMSR plates [23]. All worms were maintained at 20°C on *E. coli* strain HB101 unless indicated otherwise. The wild type strain was *C. elegans* variant Bristol, strain N2. Mutant strains used were RB754 *aak-2(ok524) X*, MR507 *aak-2(rr48) X,* KK184 *par-4(it47) V*, OD95 *unc-119(ed3) III; ltIs37 [pAA64; pie-1/mCHERRY::his-58; unc-119 (+)] IV; ltIs38 [pAA1; pie-1/GFP::PH(PLC1delta1); unc-119 (+)]*, CB1370 *daf-2 (e1370) III*, CB1372 *daf-7 (e1372) III*, CB1375 *daf-18 (e1375) IV*, CB1376 *daf-3 (e1376) X,* CF1038 *daf-16 (mu86) I*, YJ15 *ife-2 (ok306) aak-2 (rr48) X*, YJ38 *unc-119(ed3) III; ltIs37 [pAA64; pie-1/mCHERRY::his-58; unc-119 (+)] IV; ltIs38 [pAA1; pie-1/GFP::PH(PLC1delta1); unc-119(+)];aak-2(rr48)X,* YJ 18 *cep-1(ep347)I; aak-2(ok524)X*.

### Antibodies

Phospho-specific AMPK antibody (Cat. No. 2531, Cell Signaling Technology), anti actin antibody C4 (MC4, Cat. No 69100, MP Biomedicals), goat anti-rabbit (sc-2004, Santa Cruz Biotechnology; 401393-2ML, Calbiochem) and goat anti-mouse (sc-2055, Santa Cruz Biotechnology) were used.

### Western Blot Assay

Sample buffer was made and electrophoresis of proteins was performed as described [24] with the following modifications. After trans-blotting, membranes were incubated in blocking buffer (5% non-dry milk in 0.1% TBST) for 1 hour at room temperature. Membranes were incubated with antibodies at 1∶1000 dilution for the primary antibody (5% BSA in 0.1% TBST, at 4°C overnight) and 1∶4000 for the secondary antibody (5% non-dry milk in 0.1% TBST, for 1 hour at room temperature). Enhanced chemiluminescence (ECL) was used as the method of detection.

### Starvation Assay and Sample Preparation for Western Blot Assay

The worms were synchronized by egg preparation [25] and tested for starvation as described [26]. Briefly, worms were counted 24 hours after egg preparation. They were then divided between 15 ml tubes with approximately 5000 L1s in 3 ml of M9 each. Each tube was harvested on the indicated starvation day by 30 seconds of centrifugation at 1200 RPM and kept on ice for 10 min. After the supernatant was removed, samples were kept at –80°C until the western blot assay. For 24 hour adult starvation, worms were synchronized and grown on *E. coli* seeded plates until they were young adults. Well-fed worms were washed off the plates, and then washed 2 times with M9 buffer to remove remaining bacteria. Approximately 2000 adults in 3 ml of M9 buffer were incubated for 24 hours. After incubation, samples were harvested and handled as described above.

### Growth Assay

Worms were prepared and synchronized as described above. After each day of L1 starvation, approximately 100 L1s were plated on each of three *E. coli-*seeded NGM plates to grow. Every hour from 42 hours after plating, worms were examined under a dissecting microscope at 50X magnification to count worms that had molted into young adults. The numbers of worms for each time point were added up to find the time point when 50% of worms become adults.

### Oxygen Consumption Assay

Worms were prepared and synchronized as described above. To test *daf-2* and *daf-16* mutants, the synchronized population was grown at 15°C until L4, then the temperature was switched to 25°C until they became young adults without visible eggs inside. The purpose of this temperature shift was to grow *daf-2* mutants at a non-permissive temperature. To test *daf-18* mutants, the synchronized population was grown at 20°C until L4 to measure the oxygen consumption rate. Oxygen consumption rate was measured as described and normalized by the number of worms [27]. Approximately 2,000 worms were used per each sample. Every experiment was repeated at least two times with triplicates. Oxygen consumption was measured with Strathkelvin Instrument 782 system with MT200 (chamber) and 1302 Oxygen electrode. Software 782 Oxygen System Version 4.0 was used to acquire the data.

### Gonad Phenotype Assay

Worms were prepared as described above. After each day of L1 starvation (day 1 to day 7), approximately 200 L1s were plated on each of three *E. coli-*seeded NGM plates and grown to adulthood. Two days after they reached adulthood, each worm was observed under a dissecting microscope at 50X magnification to determine if it contained normal eggs and if its gonad structures were intact. The number of worms that had an abnormality in the gonad was divided by the total number of worms to calculate percent gonad defective. The average of three ‘percent gonad defective’ from triplicates measurements is shown. To measure the fertility of wild type, after 9 day starved L1s had grown to L4, 10 were transferred singly to *E. coli*-seeded NGM plates and their progeny counted. Each worm was transferred to a new plate every day to avoid being crowded and to visualize all the progeny easily. Progeny were counted three days later. Twenty worms were used to measure the sterility of *aak-2* mutants. The experiment was repeated three times.

### NaN_3_ Treatment

Worms were synchronized and grown on *E. coli* seeded plates until they were young adults. They were washed off from the plates and transferred to NGM plates containing 1 mM NaN_3_ and incubated for 6 hours. The worms were harvested for Western blot assay as described above.

### Acridine Orange Staining and Photography

500 µl of acridine orange solution (Molecular Probes Inc. A3568) at a concentration of 20 µg/ml in M9 buffer was added to a 60 mm plate with non-starved worms. The plates were put in the dark at room temperature for 1 hour. The worms were transferred to a new plate for destaining for 1 hour before images were taken. Worms were observed with a Zeiss Axioplan2 imaging microscope and pictures were taken with an AxioCam camera and Openlab software (improvision).

Statistical tests: The statistical significance of L1 longevity among mutants was tested as described [28].

## Supporting Information

Figure S1 ***daf-2*** **L1s have an increased survival span compared to wild type (+) at 25°C (a non-permissive temperature).**
(PDF)

Figure S2 **Two mutants in the insulin pathway,** ***daf-16*** **L1s and** ***daf-18*** **L1s have a short survival span compared to wild type (+).**
(PDF)

Figure S3 ***daf-7*** **mutants have normal L1 longevity at 15°C and at 22.5°C.**
(PDF)

Figure S4 ***aak-2*** **mutants have more cells in the gonad presumably due to failure of cell cycle arrest during L1 starvation.**
(PDF)

Figure S5 ***ife-2*** **mutants have increased L1 longevity.**
(PDF)

Figure S6 **A** ***daf-2*** **mutation partially rescued the L1 longevity phenotype of** ***aak-2*** **mutants.**
(PDF)

Table S1 **Post-starvation damage in gonads results in reduced brood size.**
(PDF)

Table S2 **After 5 days of starvation as L1,** ***aak-2*** **mutants develop slower than wild type.**
(PDF)
