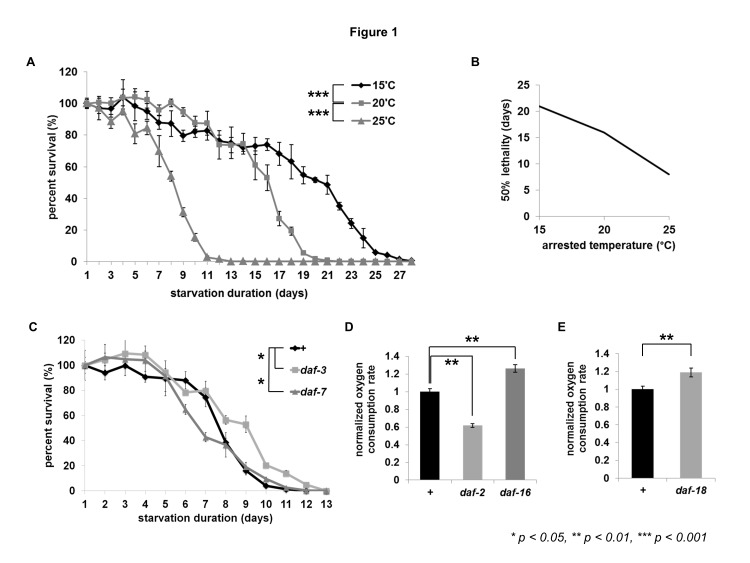# Correction: Metabolic Rate Regulates L1 Longevity in *C. elegans*

**DOI:** 10.1371/annotation/c69de5f4-dd02-4f92-9fc7-9a6a660a075e

**Published:** 2013-01-17

**Authors:** Inhwan Lee, Amber Hendrix, Jeongho Kim, Jennifer Yoshimoto, Young-Jai You

In Figure 1, the images for 1D and 1E are missing. Please see the correct Figure 1 here: 

**Figure:**