# Supplementary material for: Metabolic Rate Regulates L1 Longevity in C. elegans
Source: PLoS One. 2012 Sep 6;7(9):e44720. doi: 10.1371/journal.pone.0044720 (PMC3435313; doi:10.1371/journal.pone.0044720)

Figure S1: *daf-2* L1s have an increased survival span compared to wild type (+) at 25 ºC (a non-permissive temperature) (A) and a slightly increased survival span at 22.5 ºC (B). At a permissive temperature (15 ºC), however, there is no difference between *daf-2* and wild type (C). The results are representative of three independent experiments. *** $p < 0.001$, *NS*: not significant.

A. 25 ºC

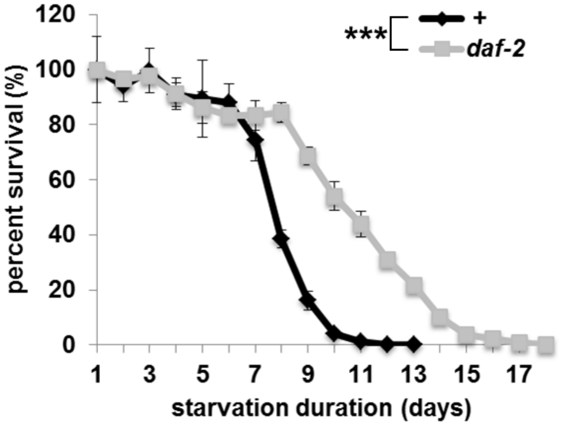

B. 22.5 ºC

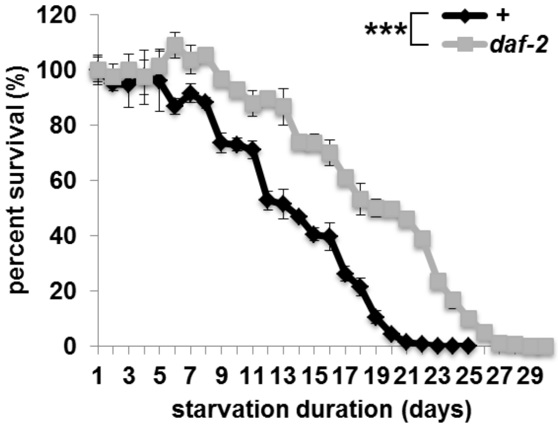

C. 15 ºC

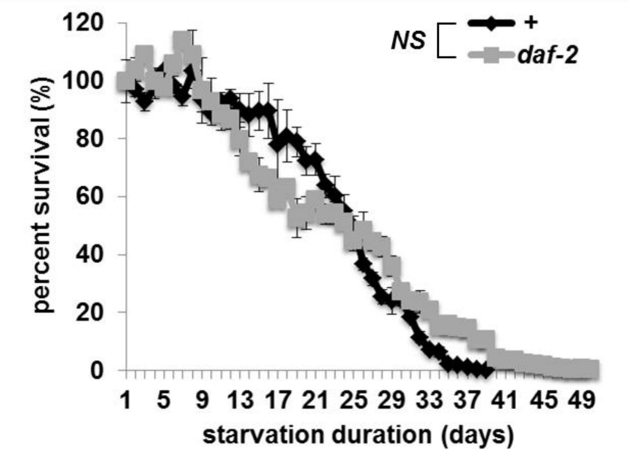

Supplement: Figure S1 — daf-2 L1s have an increased survival span compared to wild type (+) at 25°C (a non-permissive temperature). (PDF) [file pone.0044720.s001.pdf]
