# Supplementary material for: Metabolic Rate Regulates L1 Longevity in C. elegans
Source: PLoS One. 2012 Sep 6;7(9):e44720. doi: 10.1371/journal.pone.0044720 (PMC3435313; doi:10.1371/journal.pone.0044720)

**Figure S2:** Two mutants in the insulin pathway, *daf-16* L1s and *daf-18* L1s have a short survival span compared to wild type (+). The experiments were performed at room temperature (approximately at 22.5 ºC). The results are representative of three independent experiments. *** $p < 0.001$

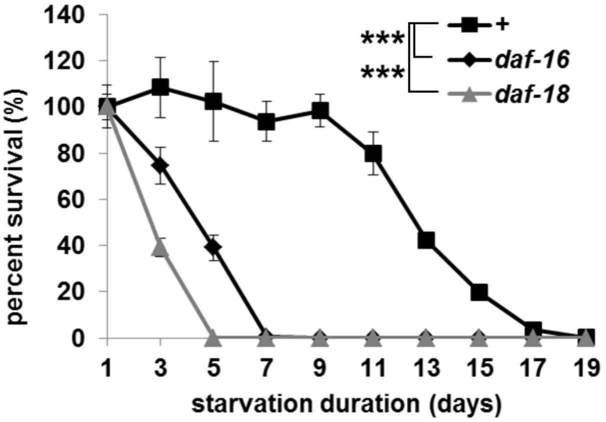

Supplement: Figure S2 — Two mutants in the insulin pathway, daf-16 L1s and daf-18 L1s have a short survival span compared to wild type (+). (PDF) [file pone.0044720.s002.pdf]
