# Supplementary material for: Metabolic Rate Regulates L1 Longevity in C. elegans
Source: PLoS One. 2012 Sep 6;7(9):e44720. doi: 10.1371/journal.pone.0044720 (PMC3435313; doi:10.1371/journal.pone.0044720)

Figure S3: *daf-7* mutants have normal L1 longevity at 15 ºC and at 22.5 ºC. The results are representative of three independent experiments. *NS:* not significant.

A. 15 ºC

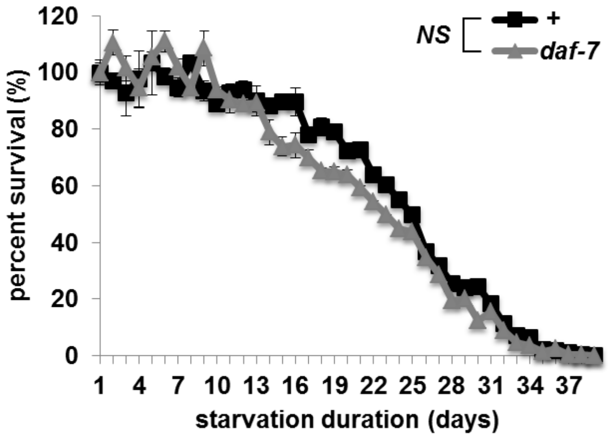

B. 22.5 ºC

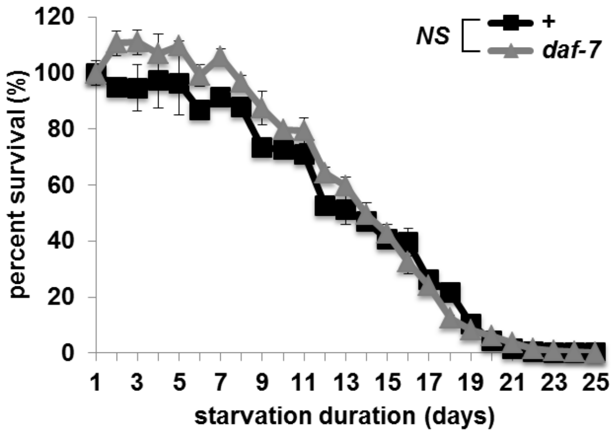

Supplement: Figure S3 — daf-7 mutants have normal L1 longevity at 15°C and at 22.5°C. (PDF) [file pone.0044720.s003.pdf]
