# Supplementary material for: Metabolic Rate Regulates L1 Longevity in C. elegans
Source: PLoS One. 2012 Sep 6;7(9):e44720. doi: 10.1371/journal.pone.0044720 (PMC3435313; doi:10.1371/journal.pone.0044720)
Supplement: Figure S4 — aak-2 mutants have more cells in the gonad presumably due to failure of cell cycle arrest during L1 starvation. (PDF) [file pone.0044720.s004.pdf]

Figure S4: *aak-2* mutants have more cells in the gonad presumably due to failure of cell cycle arrest during L1 starvation. **A.** After 5 days of L1 starvation, worms were refed to resume growth. After 12 hours of refeeding, the numbers of gonad cells that were fluorescent were counted. To visualize the germ cells, we generated *aak-2* mutants carrying a transgene of mCherry under the *pie-1* promoter (Green et al., 2011). *** $p < 0.001$ by student *t-test*. **B-D.** The increased number of gonad precursor cells was not due to fast development of *aak-2* mutants. The gonads of wild type (B) and two *aak-2* mutants, *rr48* (C) and *ok524* (D), after 5 days of starvation as L1 and subsequent 42 hours of feeding when most of wild type reaches L4. The gonads of *aak-2* mutants develop slower than wild type, suggesting that the increased number of cells in the gonad in *aak-2* mutants is not due to fast development but due to failure of cell cycle arrest during L1 starvation. **A.** Wild type gonad indicated with line. Arrow indicates the turn of the gonad. **B, C.** Gonads of *aak-2* mutants. The gonads are less developed and no turns are visible.

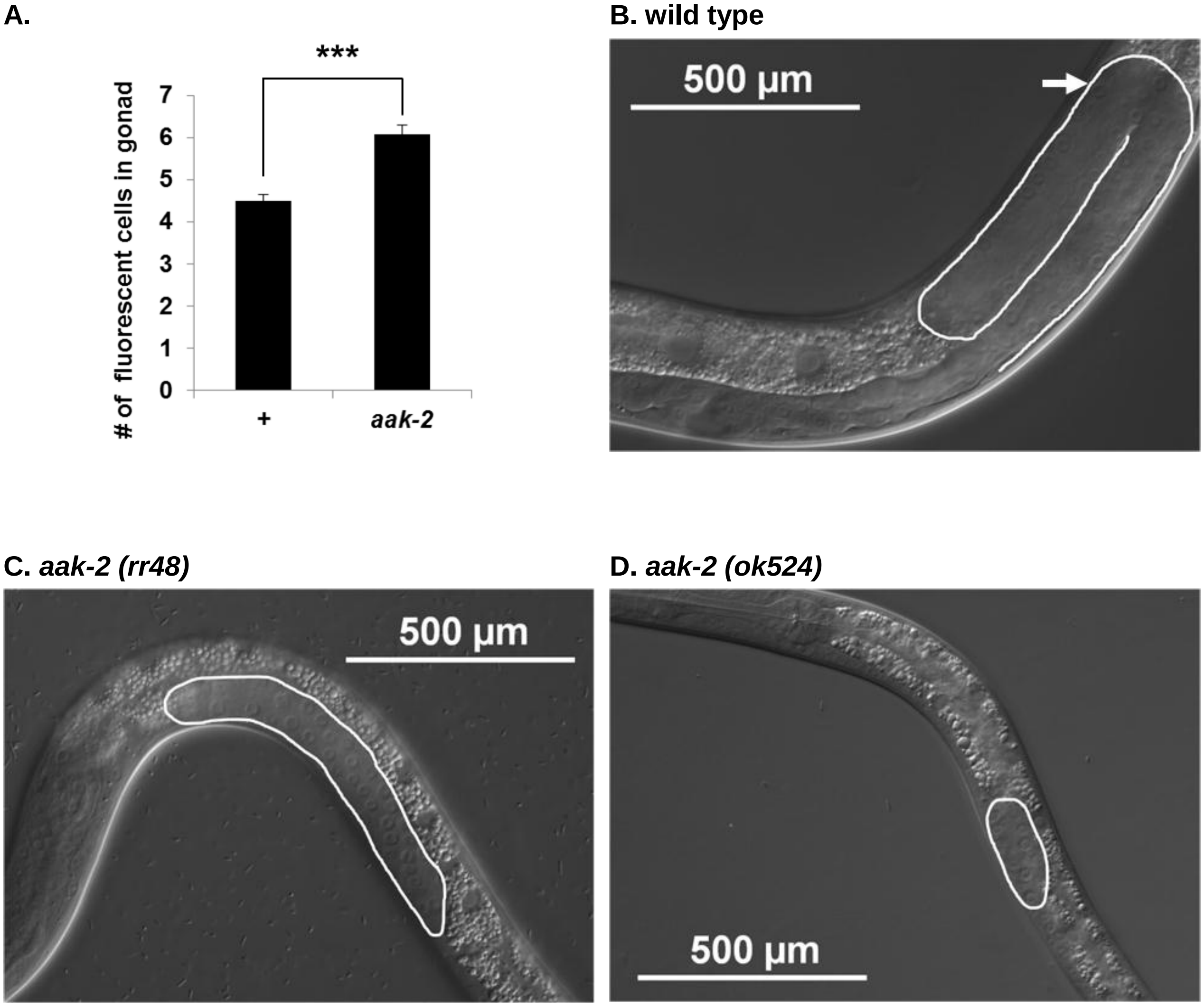

Reference:

Green, R.A., Kao, H.L., Audhya, A., Arur, S., Mayers, J.R., Fridolfsson, H.N., Schulman, M., Schloissnig, S., Niessen, S., Laband, K.*, et al.* (2011). A high-resolution C. elegans essential gene network based on phenotypic profiling of a complex tissue. Cell *145*, 470-482.
