# Supplementary material for: Metabolic Rate Regulates L1 Longevity in C. elegans
Source: PLoS One. 2012 Sep 6;7(9):e44720. doi: 10.1371/journal.pone.0044720 (PMC3435313; doi:10.1371/journal.pone.0044720)

Figure S5: *ife-2* mutants have increased L1 longevity. The experiments were performed at 22.5 ºC. The results are representative of three independent experiments. *** $p < 0.001$

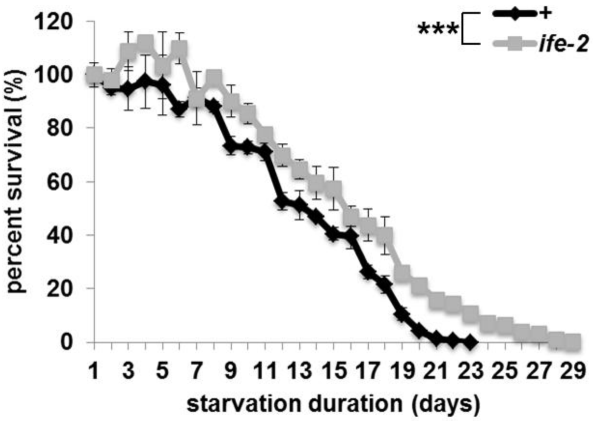

Supplement: Figure S5 — ife-2 mutants have increased L1 longevity. (PDF) [file pone.0044720.s005.pdf]
