# Supplementary material for: Metabolic Rate Regulates L1 Longevity in C. elegans
Source: PLoS One. 2012 Sep 6;7(9):e44720. doi: 10.1371/journal.pone.0044720 (PMC3435313; doi:10.1371/journal.pone.0044720)

Figure S6: A *daf-2* mutation partially rescued the L1 longevity phenotype of *aak-2* mutants. The experiments were performed at 25 ºC, a non-permissive temperature for *daf-2*. The results are representative of three independent experiments. *** $p < 0.001$

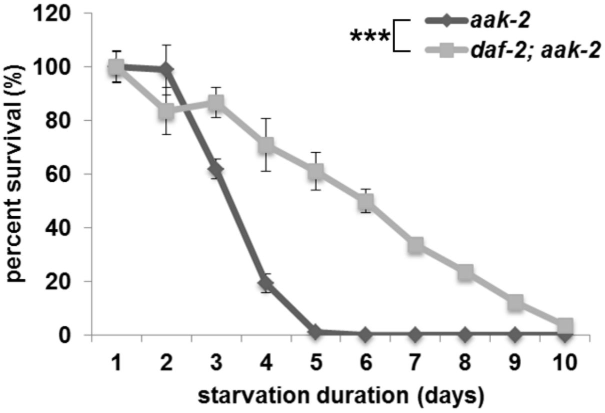

Supplement: Figure S6 — A daf-2 mutation partially rescued the L1 longevity phenotype of aak-2 mutants. (PDF) [file pone.0044720.s006.pdf]
