# Supplementary material for: Metabolic Rate Regulates L1 Longevity in C. elegans
Source: PLoS One. 2012 Sep 6;7(9):e44720. doi: 10.1371/journal.pone.0044720 (PMC3435313; doi:10.1371/journal.pone.0044720)
Supplement: Table S1 — Post-starvation damage in gonads results in reduced brood size. (PDF) [file pone.0044720.s007.pdf]

Table S1

starvation days	number of progeny	
	from mothers with damaged gonads	from mothers with normal gonads or less damaged gonads
**1**	N/A	272 ± 8.15***
**9**	1.67 ±1.04***	141.4 ± 20.41***

Table S1: Post-starvation damage in gonads results in reduced brood size.
9 days of starvation as L1 induces gonad damage once L1s grow to adults and results in reduced brood size. For each group 15 mothers were tested (see Materials and Methods for detail). Numbers are mean ± S.E.M. All three results are statistically different from each other. *** $p < 0.001$ (Mann-Whitney U Test)
