# Supplementary material for: Metabolic Rate Regulates L1 Longevity in C. elegans
Source: PLoS One. 2012 Sep 6;7(9):e44720. doi: 10.1371/journal.pone.0044720 (PMC3435313; doi:10.1371/journal.pone.0044720)
Supplement: Table S2 — After 5 days of starvation as L1, aak-2 mutants develop slower than wild type. (PDF) [file pone.0044720.s008.pdf]

Table S2

genotypes	young adults	≤ L4s	total
**+**	119	59	178
aak-2 (rr48)	0	205	205
aak-2 (ok524)	0	286	286

Table S2: After 5 days of starvation as L1, *aak-2* mutants develop slower than wild type. L1s were starved for 5 days then fed to grow. After 52 hours of feeding, the gonad development was used to confirm adulthood. Neither of two different *aak-2* alleles reached adulthood after 52 hours of feeding from L1s, whereas about 67% of wild type reached adulthood. The result is representative of three independent experiments.

Reference:
